# The Effect of High Pressure Techniques on the Stability of Anthocyanins in Fruit and Vegetables

**DOI:** 10.3390/ijms18020277

**Published:** 2017-01-27

**Authors:** Krystian Marszałek, Łukasz Woźniak, Bartosz Kruszewski, Sylwia Skąpska

**Affiliations:** Department of Fruit and Vegetable Product Technology, Prof. Wacław Dąbrowski Institute of Agricultural and Food Biotechnology, 36 Rakowiecka Street, 02-532 Warsaw, Poland; lukasz.wozniak@ibprs.pl (Ł.W.); bartosz.kruszewski@ibprs.pl (B.K.); sylwia.skapska@ibprs.pl (S.S.)

**Keywords:** anthocyanins, stability, degradation, high pressure processing, high pressure carbon dioxide, high pressure homogenization

## Abstract

Anthocyanins are a group of phenolic compounds responsible for red, blue and violet colouration of many fruits, vegetables and flowers. The high content of these pigments is important as it influences directly their health promoting properties as well as the sensory quality of the product; however they are prone to degradation by, inter alia, elevated temperature and tissue enzymes. The traditional thermal methods of food preservation cause significant losses of these pigments. Thus, novel non-thermal techniques such as high pressure processing, high pressure carbon dioxide and high pressure homogenization are under consideration. In this review, the authors attempted to summarize the current knowledge of the impact of high pressure techniques on the stability of anthocyanins during processing and storage of fruit and vegetable products. Furthermore, the effect of the activity of enzymes involved in the degradation of these compounds has been described. The conclusions including comparisons of pressure-based methods with high temperature preservation techniques were presented.

## 1. Introduction

Consumption of fruit and vegetable products containing numerous bioactive compounds can significantly reduce the risk of various degenerative diseases. Much current research has been carried out on the content of different phytochemicals and their positive health effect. It has been demonstrated that a diet rich in fruit and vegetables is the source of polyphenols (phenolic acids, flavonols, catechin monomers, proanthocyanidins, flavones, flavanones and anthocyanins) [1]. From the consumer’s point of view anthocyanins play the particularly important role because of their health promoting properties and impact on a key quality parameter, colour, influencing the sensory acceptance of the product.

**Figure 1:**
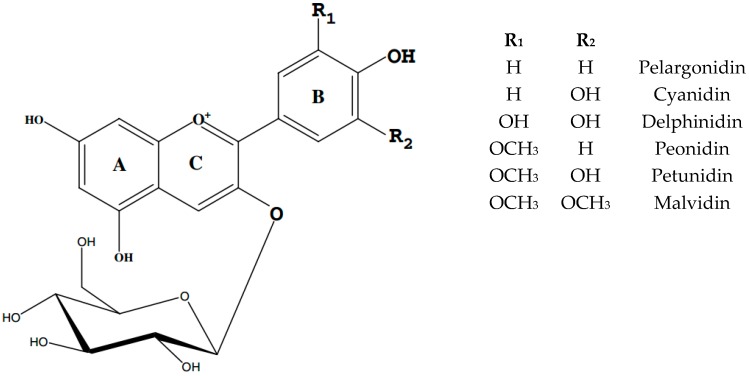
Structure of anthocyanins [5].

Anthocyanins are the largest and the most diverse group of plant pigments responsible for the red, violet and blue colours of various fruit and vegetables [2,3,4,5]. These pigments are water-soluble phenolic compounds, located in vacuoles and part of a large group of flavonoids that have a common C_6_–C_3_–C_6_ structure consisting of two aromatic rings linked through an oxygenated heterocycle (Figure 1). The name of anthocyanins describes both aglycones (anthocyanidins) as well as their glycosides. While there are six common anthocyanidins found in plant tissues, more than 540 anthocyanin pigments have been identified in nature [6], with most of the structural variation coming from glycosidic substitution at the 3 and 5 positions and possible acylation of sugar residues with organic acids causing differences in their colour and stability [2,6]. Increased glycosidic substitution, and in particular, acylation of sugar residues, will increase pigment stability [6].

Polyphenols, especially anthocyanins, are effective antioxidants with a beneficial effect on human health and disease prevention. They can promote healthy vision, dermal health, have a positive effect on the cardiovascular system as well as exhibit neuroprotective, anticarcinogenic and antidiabetic properties [6,7,8]. Dietary consumption of anthocyanins, especially from red fruits, certain vegetables or red wines should be as high as 200 mg per day. The “French Paradox” has been explained by the high consumption of resveratrol and other polyphenols, such as anthocyanins with red wine as a part of a diet rich in saturated fats.

Reduction of losses of anthocyanins is the target for food production. However, to achieve any biological effect in the human body, anthocyanins must be bioavailable, i.e., effectively absorbed from the gastrointestinal tract into the circulation and delivered to the appropriate location within the body. Studies of the oral intake of anthocyanins-rich fruits have confirmed increased antioxidant status of the serum [9,10], despite very low bioavailability of these compounds (1%–4% of dose uptake) [5,11,12].

Many foods that contain anthocyanins are thermally processed prior to consumption and this process can significantly influence the degradation of anthocyanins and vitamin C content in the final product. The degradation level of anthocyanins may be as high as 50%, depending on the temperature and the heating time [4,13]. Thermal processing implies heating up to 50–150 °C, depending on the pH of the product and the desired shelf life. Consumer demand for nutritious foods, which are minimally processed, without using artificial preservative substances, has led to an interest in non-thermal technologies, such as high pressure processing, high pressure carbon dioxide or high pressure homogenization [14]. These techniques, based on elevated pressure, are employed at ambient or mild temperatures, thereby avoiding negative thermal effects on food nutritional and sensorial quality parameters [15].

The present review will summarize and critically evaluate recent studies on the effect of high pressure techniques on the anthocyanin content in fruit and vegetable products. Particular focus will be given to the degradation of anthocyanins subjected to these techniques with the special consideration for the kinetics and mechanisms of these reactions, including enzymatic decomposition.

## 2. The Principles and Food Applications of High Pressure Techniques

### 2.1. High Pressure Processing (HPP)

High hydrostatic pressure processing (HHP), high pressure processing (HPP) or ultra-high pressure (UHP) is a non-thermal technique used in food preservation, starch gelatinization, enzyme inactivation, osmotic drying enhancement, pressure-shift freezing and thawing enhancement [3,4,16,17,18]. HPP is derived from material science in which samples are treated with pressures above 100 MPa [3] and is an interesting alternative to traditional food preservation [19]. High pressure affects noncovalent bonds (such as hydrogen, ionic, and hydrophobic bonds) substantially as such bonds are usually very sensitive to pressure. The low molecular weight food components (those responsible for nutritional and sensory characteristic) are not affected by pressure whereas high molecular weight components (whose tertiary structure is important for functionality determination i.e., proteins) are pressure sensitive [8,16].

The application of high pressures to food preservation started around 1900 when the Hite group investigated its effect on milk, meat, fruits and vegetables at pressures up to 650 MPa and noted significant reduction of microbes [18]. The first commercial products processed by hydrostatic pressure were launched in Japan in 1990. Since then, researchers and industrial companies have explored this technique, especially in Japan, Europe and US.

There are two major limitations to the further development of HPP. First, the database of pressure processing kinetics parameters of microbial and enzyme inactivation, such as *D*-value and *z*-value, is not comprehensive enough to ensure the reliability of HPP as an alternative to thermal processing. Secondly, developing a continuous pressure food processor remains an engineering challenge [18]. The highest advantages of HPP techniques are the fresh taste and natural colour of preserved products.

### 2.2. High Pressure Carbon Dioxide (HPCD)

HPCD is a method of cold pasteurization that inactivates food microorganisms and tissue enzymes through the combined effect of high pressure, mild heat and the ability of carbon dioxide to penetrate food samples. This technology is already over 50 years old, however so far it has not been implemented in large-scale food industry processes. Similarly to HHP, this technique is described by several names, including Dense Phase Carbon Dioxide (DPCD) and, when used under specific pressure and temperature conditions, Supercritical Carbon Dioxide (SCCD).

The microbial inactivation during the HPCD processing is usually connected with lowering pH inside the cell, physical disruption of microbiota, modification of cell membranes and extraction of intracellular compounds. The rewarding decontamination effect is possible to achieve with minimum tenfold lower pressure compared to pressure used in HPP. The most important advantages of this technique is the possibility of microbial and tissue enzymes inactivation under much lower pressures compared to HPP technique. Still, the exact mechanism of enzyme inactivation still remains unclear; one of the main hypotheses describes the conformational changes of the enzymes.

Several HPCD treatment systems have been developed. In batch systems samples are placed inside a thermostated pressure chamber, CO_2_ is pumped in and left for some period of time to ensure penetration. The continuous systems include membrane contractors, micropore filters or a continuous flow of the sample and carbon dioxide. HPCD is usually tested for use in liquid foods, such as fruit juices, beer and milk. Treatment of whole fruits was unsuccessful due to severe tissue damage [20].

### 2.3. High Pressure Homogenization (HPH)

HPH is a new technology developed as a result of food producers’ need to obtain new quality products with increased or well preserved pro-healthy compounds, such as vitamins and polyphenols [21,22,23,24,25,26]. It is based on the same principle as traditional homogenization but employs 10–15 times higher pressures within a range from 100 up to 400 MPa. The process carried out at upper pressure range ≥200 MPa is called an ultra-high pressure homogenization (UHPH) [25,26,27].

The principle of HPH is forcing the pressurized liquid through a gap of a few micrometers in width (disruption valve), where it is submitted to rapid acceleration. The resulting pressure drop simultaneously generates intense fluid-mechanical stresses such as local cavitation, shear stress, collision, and turbulence which lead to particle comminution [21,28,29]. For this reason, HPH is a suitable and effective technology for the continuous dynamic production of fluid like and pumpable foods. The main industrial application of HPH is the production of stable vegetable and livestock milk emulsions and their by-products [26,29,30,31,32]. This non-thermal technology is also investigated for the production of high quality fruit and vegetable juices and beverages [23,25,33,34,35,36]. The biggest advantages of HPH in cloudy juices are simultaneous microbial preservation and the stabilization effect. Other industries with high applicability potential are cosmetology and pharmaceutics [37,38].

Reducing the particles and emulsion droplets to finer dimensions modifies the physical characteristics of liquid or semi-liquid mixtures, such as the viscosity and mean particle size of the suspended solids, which affect texture and promote freshness attributes [21,33]. High pressure present during homogenization, especially above 200 MPa (UHPH) has been shown to have great results in the reduction of pathogenic and spoilage microorganisms by microbial cell disruption without decreasing the nutritional value of processed food products [29,33,36,39,40,41]. Studies have shown that the level of inactivation of the microbial load depended on the applied pressure, number of HPH passes, inlet and valve temperatures, matrix type and the nature of microflora [31,33,36,42,43,44]. The most sensitive to HPH are yeast and moulds, more robust are gram negative bacteria, while gram positive bacteria are the most resistant [31,32,45,46].

In general, raising the homogenization pressure and inlet/valve temperatures significantly increased the reduction of microorganisms. However, with increasing pressure the temperature in the valve also increases (adiabatic heating about 0.15–0.20 °C/MPa), so it is important to monitor and adjust both parameters not to generate excessive heating with a negative impact on nutritional value and the loss of biomolecule functionality of processed foods [22,25,28,33]. In most cases, efficient, rapid cooling systems were installed after the homogenizer valve [26,34,35,47]. The effectiveness of HPH treatment for given pressure can be improved by performing multiple passes [36,43,45,46]. Some authors have found a first order inactivation kinetics as a function of the number of passes [45,46].

## 3. Anthocyanins Stability Factors

### 3.1. Role of Enzymes in Anthocyanins Degradation

Enzymes, such as β-glucosidase (β-GLC), polyphenoloxidase (PPO) and peroxidase (POD), are mainly responsible for the degradation of anthocyanins. The anthocyanin-degrading enzymes may be native, present in the plant tissue, or they may result as a product of microbial contamination [6]. Another possible source is the side activity of commercial enzyme preparations used for technological reasons during industrial juice extraction.

The search of anthocyanins-degrading enzymes is performed on postharvest fruit and vegetables, during processing and storage. Intercellular decompartmentation and cell layer reparation begins during storage, and the pigments may be exposed to microenvironmental conditions that differ from those in growing plants, including enzymes that are not located in the vacuoles when the plant cells are intact [2].

β-GLC (EC 3.2.1.21) influences directly the anthocyanins, but the action of PPO (EC 1.14.18.1) and POD (EC 1.11.1.7) is indirect [6]. β-GLC catalyzes the hydrolysis of aryl or alkyl β-d-glucosides and is involved in the liberation of aglycones from non-volatile glucosides [48]. These enzymes catalyze the loss of glycosidic moiety leading to the formation of anthocyanidin consequently affecting product colour. Specificity of β-glucosidase leads to a reduction of anthocyanins containing glucose in their structure because of the greater affinity towards glucose compared to other sugars [3]. PPO and POD are the principal enzymes involved in the enzymatic browning of plant tissues. PPOs are copper-containing isoenzymes responsible for the hydroxylation of monophenols to *o*-diphenols (by monophenol oxidase) and the oxidation of diphenols to quinones (by diphenol oxidase) which in the presence of proteins form complex brown pigments [49,50]. POD carries a “b”-type heme as a prosthetic group and, as another redox enzyme, participates in several plant metabolic processes such as the catabolism of auxins, lignification of the cell wall, as well as browning reactions, resulting in products which participate in the oxidation of various electron donors with H_2_O_2_ [51].

**Figure 2:**
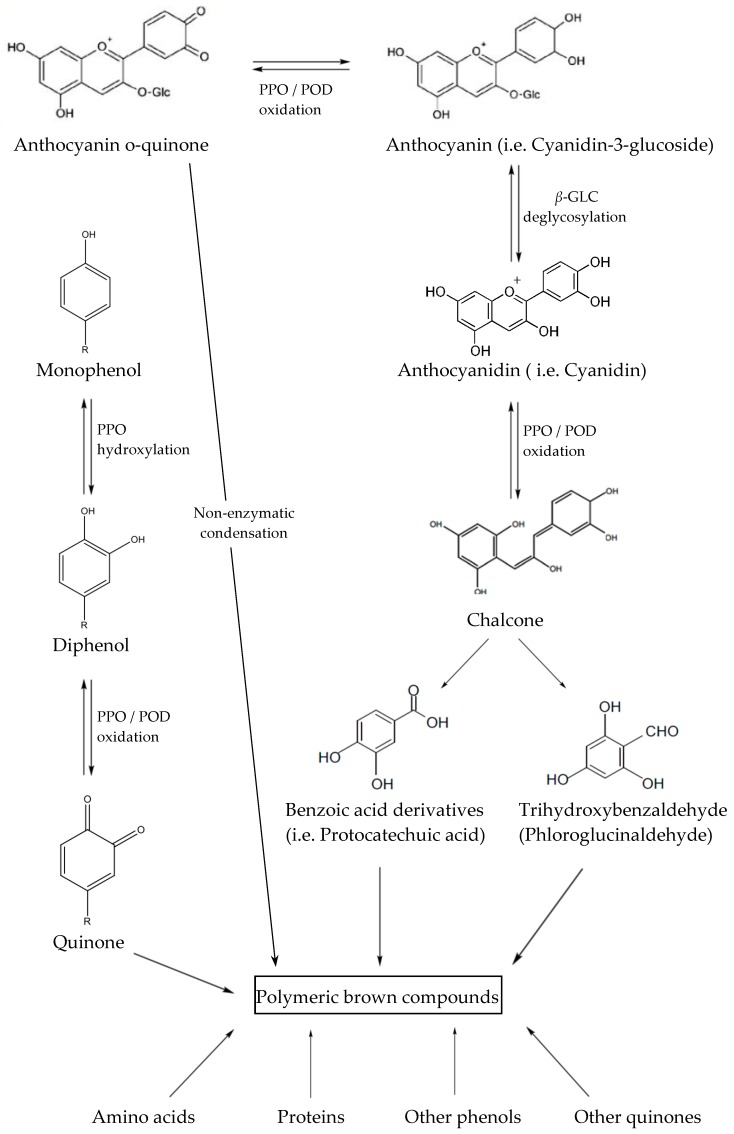
Scheme describing anthocyanins degradation. PPO: polyphenol oxidase, POD: peroxidase, β-GLC: beta glycosidase.

It was proposed that anthocyanins are firstly hydrolyzed by the β-glucosidase, forming anthocyanidins which can be oxidized by polyphenol oxidase and/or peroxidase (Figure 2). PPO at the presence of oxygen can accelerate the enzyme degradation of anthocyanins. Oxidation of simply phenols, resulting from PPO activity, leads to the formation of the corresponding o-quinones, which may then accelerate anthocyanins degradation in the oxidative reaction. These quinones react with anthocyanins to form brown condensation products [4]. This observation confirms that PPO plays a vital role in anthocyanins degradation. Peroxidases also degrade anthocyanins in fruit and vegetable products provoked by the H_2_O_2_ present in plant cells [2,4]. The other pathway of anthocyanin changes in fruit and vegetable products are non-enzymatic chain reactions due to hydrolysis of glucosidic linkage to form aglycone or hydrolytic cleavage of pyrilium ring to form substituted chalcones. Thermal degradation is assumed to be a hydrolytic reaction and availability of water is essential for anthocyanins degradation [52].

Pressure techniques can influence the enzyme activity responsible for anthocyanins stability. The inactivation effect depends on many factors, such as pressure, temperature and processing time as well as the physicochemical properties of raw materials such as total soluble solids and pH [3,48]. From the industrial point of view it is important to care about reduction as many factors accelerating enzymatic reaction as possible, i.e., oxygen or temperature.

### 3.2. Other Factors Affecting Anthocyanin Stability

Anthocyanins are very susceptible to degradation not only through the activity of tissue enzymes but through other factors such as temperature, light, pH, sugars, presence of oxygen, sulphites, ascorbic acid, metal ions and co-pigments [18,52,53,54]. The stabilization of anthocyanins in different mixed products can be achieved by the increasing of acidity for example by carefully composing recipes or addition of organic acids. The colour and stability of anthocyanin solutions are dependent on equilibrium of 4 species due to changes in pH, protonation or hydratation reactions. Anthocyanins exist primarily in the form of red flavylium cations at pH < 3. As the pH is raised, kinetics and thermodynamic competition occurs between the hydration reaction on position 2 of the flavyium cation and the proton transfer reaction related to its acidic hydroxyl groups. This flavylium cation converts into purple-violet quinonoidal bases as pH increases to 4. At pH 4–6, nucleophilic attack by water at C-2 yields colorless hemiketal forms, which can undergo ring opening to yield yellow retro-chalcones [52,55]. The degradation of anthocyanins is also affected by solution composition and environmental conditions such as oxygen concentration [56]. The oxygen is an important factor in enzymatic reactions described earlier.

On the other hand, the stability of anthocyanins is increased by copigmentation that occurs in the presence of certain compounds such as other phenolics and metal ions such as magnesium, copper and aluminium [53]. Anthocyanins under different processing and storage conditions readily convert into insoluble polymeric brown pigments. This pigments also rapidly degrade at elevated temperature due to their high reactivity causing monomeric anthocyanins to polymerize forming brown compounds [52].

The structure of anthocyanins plays a crucial role in their stability. In general, the presence of hydroxyl groups (–OH) decreases stability of the molecule, while the occurrence of methoxy groups (–OCH3) increases it [57]. The stability of anthocyanins is also increased by the bigger number of sugar moieties and their acylation by aliphatic and cinnamic acids [7,52,58]. In nature, the hydroxyl groups of the substituted glycosyls of anthocyanins are typically acylated by organic acids via ester bonds, which is referred to as anthocyanins glycosyl acylation, to yield acylated anthocyanins. Anthocyanin glycosyl acylation affects their chemical stability anthocyanins. Glycosyl acylation is one of the key factors for creating diversity within anthocyanins molecules and the types, numbers and linkage position of the acyl groups substantially increase the types of anthocyanins and their stability [59].

The stability can be also increased by the formation of addicts made of two molecules of anthocyanins (self-association) or anthocyanin and other compounds (co-pigmentation). The copigmentation occurs most often between anthocyanin and other phenolics like hydroxycinnamic acids [53].

## 4. Inactivation of Enzymes Responsible for Degradation of Anthocyanins during Pressure Processes

### 4.1. High Pressure Processing (HPP)

High pressure can have an influence on the biochemical reactions, since it is able to change the quaternary structure of proteins. The effect of HPP on the proteins/enzymes is reversible up to 400 MPa and is linked with the conformational changes, subunit dissociation and association process [60]. High pressure treatment generally leads to a reduction of the fruit and vegetable tissue enzymes activity [61,62,63,64,65,66,67], but there are also some results proving that pressure can increase the activity of enzymes [68,69,70]. A significant decrease of selected enzymes activity were noted in fruits such as: apples, avocado, grapes, pineapple, strawberry, raspberry and feijoa under a pressure range from 400 to 900 MPa, depending on food composition and pH. At a lower pressure range from 50 to 400 MPa some authors observed even enhanced enzyme activity [66,67]. Moreover, higher pressure induced inactivation of tissue enzymes was found at a lower pH [71]. Apart from the pH, pressure inactivation was also influenced by the addition of salts, sugars and other compounds such as benzoic acid, glutathione or calcium chloride [72]. The application of pressure under commercial units allowed to complete the inactivation of enzymes only in a combination with mild temperature (~50 °C). The influence of HPP on three of the most important enzymes responsible for anthocyanins degradation found in fruit and vegetables, polyphenol oxidase (PPO), peroxidase (POD) and β-glucosidase (β-GLC), was presented in Table 1. Generally speaking the application of HPP allows only to decrease the activity of selected tissue enzymes. Complete inactivation is possible but with pressure over the pressures achievable in industrial applications. The use of lower pressure can be compensated by longer time of the process, which however may significantly decrease the efficiency of production.

### 4.2. High Pressure Carbon Dioxide (HPCD)

The impact of dense phase carbon dioxide on the activity of tissue enzymes in strawberry juice has been reported in two works of Marszałek et al. [85,86]. Authors tested influence of HPCD at pressure up to 60 MPa, time up to 30 min and temperature up to 65 °C. It was shown that activity of polyphenol oxidase was fully inhibited after treatment at the temperatures of 45 and 65 °C with the use of pressures of 10–60 MPa and treatment times of 10–30 min. On the other hand, no full inactivation of peroxidases has been achieved at these conditions; the best results (reduction to <10% of initial activity) were observed for samples processed at 65 °C, regardless of pressure and time. The activity of enzymes has not changed significantly during the 12 weeks of storage of the processed juice. The team led by Gui [87] has been focusing on the impact of supercritical carbon dioxide on native polyphenol oxidase in cloudy apple juice. The maximum reduction of activity was more than 60% after processing at 55 °C and 30 MPa for 60 min, whereas Del Pozo-Insfran et al. [88] noted ca. 40% inactivation of the same enzyme in muscadine grape juice treated by dense phase carbon dioxide at pressure up to 48 MPa. The impact of HPCD on the quality of carrot juice was investigated by Zhou et al. [89]. The researchers used a batch system to treat juice at a temperature of 25 °C, pressure of 10–30 MPa and treatment times of 15–60 min and found out that residual activity of polyphenol oxidase was about 2%–4% of the initial one. Spilimbergo with co-workers dealt with HPCD preservation of fresh-cut carrot slices [90]. They used pressure of 8 and 12 MPa, temperature from 22 to 45 °C and processing time 5–45 min. The full inactivation of tissue enzymes has not been achieved, however a 70% reduction of activity after the treatment was observed. Another report by Niu et al. [91] was focused on apple slices. The scope of work included experiments at a pressure of 20 MPa, temperature range 25–65 °C; each of the processes was 20 min long. The experiments showed that in all tested parameters carbon dioxide treatment was able to fully suppress the activity of PPO in apples. The possibility of inactivation of tissue enzymes by HPCD is significantly higher compared to traditional HPP technique probably due to decreasing of pH of treated sample. In many cases, the total inactivation of selected enzymes in acidic products was achieved at minimum tenfold lower pressures compared to HPP. Pressurization with carbon dioxide causes significant changes of pH due to formation of carbonic acid, influencing on the enzyme activity. Changes in enzyme activity under HPCD may be also attributed to changes in protein structure, enzyme stability and/or disruption of enzyme-substrate interactions under the conditions of processing [88]. Some authors connected lowering of enzyme activity with changes in secondary structure (enzyme conformation) that was dependent on the kind of source [87,88]. Alternatively, the pH alternation by carbon dioxide might be associated with enzyme-bound arginine reactions to form a bicarbonate-protein complex that was potentially responsible for the decreasing of enzyme activity [88]. Further research is desirable in this field as well as the elaboration of relative industrial appliances for preservation of food products using HPCD.

### 4.3. High Pressure Homogenization (HPH)

Residual endogenous enzymes activity like PPO or POD in particular fruit or vegetable products treated with HPH can vary, and depend on the device type, processing method, especially the number of steps, their duration, and heating temperature [33]. In addition, pretreatment operations like thawing and blanching can markedly affect the enzymes activity, as well as anthocyanins content [92,93].

Within the context of bioactive compounds retention, native enzymes inactivation is carefully studied in terms of application of HPH in food processing. So far, reports on HPH inactivation of enzymes which are related to food quality concerned: pectin methylesterase (PME), polygalacturonase (PG), pectate lyase (PL), peroxidase (POD) and polyphenol oxidase (PPO) [21,25,33,34,35,38,43]. Enzyme activity reduction by HPH is acquired by a combination of set temperature and pressure [27,28]. Inlet temperature and holding time of liquid at outlet temperature are very important [41,94,95], but pressure has the main impact on enzyme inactivation. Due to the mode of applied pressure, fluid-mechanical stresses are changing resulting in local cavitation, shear stress, collision, and turbulence, which affect the structure of the enzyme protein and its substrates [96,97]. As a result of dimensional configuration changes, the active site of enzymes lose their activity; moreover unfolded molecules have a tendency to aggregate, which also lead to their inactivation [33,47,98]. Aside from HPH processing parameters, specific characteristics of a particular enzyme are very important. In food, they occur in several forms (isozymes), which have various sensitivities to temperature, pressure and fluid-mechanical stresses [41,98]. Ripeness and matrix composition such as medium, presence of other proteins, sugars or salts and pH have an influence on thermo- and pressure-lability of enzymes [95,99].

Reported enzyme activity reduction by HPH varied but generally increased with preliminary warming of food to higher temperatures, increasing pressure and number of passes through the high pressure (HP)-valve [95,98,100]. Enzyme inactivation at HPH-treated (<200 MPa) juices was usually insufficient [41,98]. However, juice samples after treatment were more stable than the control samples [100,101]. UHPH processing (≥200 MPa) was more effective in this matter. In some cases the obtained activity reduction was in the range of 90%–100% [24,41]. However, even UHPH treatment did not always guarantee sufficient enzymes inactivation in processed food [95,100,102,103].

## 5. Stability of Anthocyanins under Pressurization

### 5.1. High Pressure Processing (HPP)

**Figure 3:**
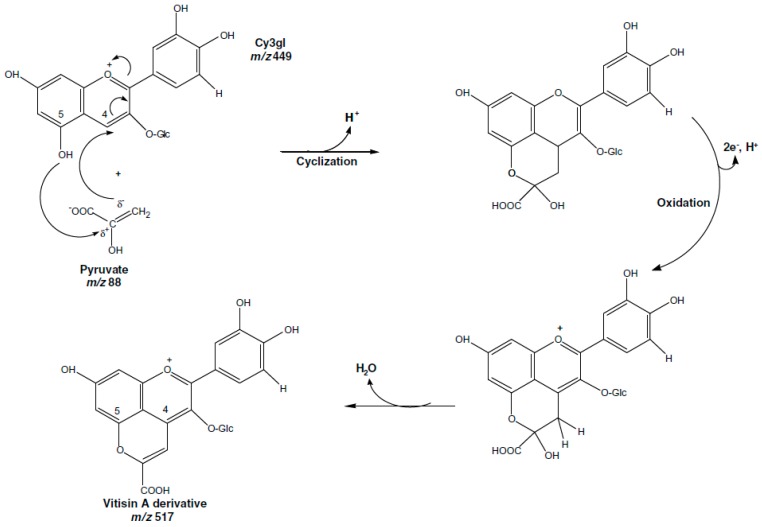
Condensation reaction of cyanidin-3-glucoside and pyruvate at 600 MPa and 70 °C proposed by Corrales et al. [106].

HPP preservation (at low, room and mild temperature) has a limited effect on anthocyanins degradation compared to thermal processing. Pelargonidin-3-glucoside and pelargonidin-3-rutinoside in raspberries and strawberries processed at 800 MPa and 18–22 °C for 15 min were stable [74], whereas some authors detected slight changes of anthocyanins in different red fruits and products obtained therefrom under HPP at mild and high temperatures (Table 2) [64,76,104,105]. Corrales et al. [106] and Barba et al. [107] reported insignificant changes of anthocyanins processed by HPP up to 600 MPa at room temperature, whereas increasing the temperature up to 70 °C caused 25% losses of this pigments. This degradation was ca. 20% higher compared to the traditional thermal treatment at the same temperature, indicating that HPP coupled with higher temperatures accelerates anthocyanins decomposition [3]. These changes are mostly attributed to the conversion of monomeric anthocyanins to more condensed compounds during storage. This condensation reaction (Figure 3), induced by high pressure and/or temperatures, involves covalent association of anthocyanins with other flavonols or organic acids leading to the formation of a new pyran ring by cycloaddition. Anthocyanin condensation is responsible for the changes of colour i.e., in red wine during storage, by forming complexes of brown pigments [106]. It implies that high pressure should be used taking into account the negative influence of the synergistic effect of pressure and temperature. Further works on this technique should consider the calculation of heat dose as a function of time and process temperature.

The kinetics of anthocyanins degradation under HPP was investigated by Verbeyst et al. [7]. The authors described degradation of anthocyanins under thermal processing (TP) and HPP as being well described by a first-order kinetic model. They concluded that the higher temperature used under TP caused faster anthocyanins degradation. The reaction rate constant increased with increasing the pressure as well as the temperature rising under HPP. The kinetic rate constant noted for anthocyanins degradation generally increased with the increasing the pressure as well as temperature and ranged from 0.7 to 7.1 × 10^−2^ min^−1^.

The activation energy (*E*_a_-value) under high pressure coupled with temperature from 80 to 110 °C ranged from 58 to 63 kJ/mol whereas this value calculated for TP at atmospheric pressure in the range from 95 to 130 °C was 94 kJ/mol. This means that the reaction rate constant at atmospheric pressure is less sensitive on temperature compared to reaction rate constants at similar temperatures under high pressure. The pressure dependence of the degradation rate constant was described by Eyring equations and expressed as activation volume (*V*_a_-value). The values of the activation volume were negative, indicating an accelerating effect of pressure on the degradation of anthocyanins. As these values were small, the pressure dependence of the reaction rate constants was limited [67,75].

The biggest advantages of HPP are generally insignificant changes of anthocyanins concentration at cold and room temperature under pressures up to 600 MPa. Unfortunately, for the efficient tissue enzyme inactivation mild heating is required. The increasing of temperature of HPP over 50 °C accelerates thermal degradation and condensation reaction of these pigments. Despite this, degradation of anthocyanins under HPP connected with moderate temperature is much lower compared to the traditional batch pasteurization.

### 5.2. High Pressure Carbon Dioxide (HPCD)

The impact of high pressure carbon dioxide on the quality of strawberry juice during the batch operation has been investigated by Marszałek et al. [85] at pressure, temperature and time up to 60 MPa, 65 °C and 30 min, respectively. In this study, they reported that no significant losses of anthocyanins were observed after 30 min of preservation at the temperature of 45 °C and pressures of 30 MPa and 60 MPa. Subsequent work of this team [86] expanded the range of conditions examined; time of preservation was 10–30 min, temperature and pressure of the process were 35–65 °C and 10–60 MPa, respectively. The maximal reduction of anthocyanin content (ca. 10%) was observed for the longest time and most harsh conditions; for the majority of combinations of parameters changes of anthocyanin concentrations was insignificant.

Stability of anthocyanins in juice from blood oranges was tested by Fabroni et al. [110]. They applied three variants of the continuous process. Only the process carried out at 36 °C, 230 bar and 0.770 g of carbon dioxide per g of sample showed significant degradation of pigments. Other variants using lower pressure (130 bar) and/or reduced CO_2_/juice ratio had no significant impact.

The team led by Ramirez-Rodrigues [111] was investigating the impact of SCCD on *Hibiscus sabdariffa* beverage in a continuous process. Only one set of working conditions was tested: 30.6 MPa, 8% CO_2_, 40 °C and treatment time 6.8 min. They observed a small (ca. 3%), but significant degradation of anthocyanins.

Zou et al. [112] were dealing with the effect of HPCD on mulberry juice. Samples were treated in a batch process for 10 min at a temperature of 55 °C and pressure of 15 MPa. Surprisingly the increase of anthocyanin content of about 16% has been observed. The authors linked this phenomenon with a liberation of these compounds from the tissue, however this finding has not yet been confirmed by other researchers.

Del Pozo-Insfran et al. [113,114] concentrated their work on the preservation of muscadine grape juice. They carried out the process continuously at pressure of 34.5 MPa with 8% and 16% of CO_2_ (no information about temperature of process was provided). The preservation process has not changed the content of anthocyanins in a significant way in neither of the tested conditions. 

The significant advantage of HPCD over HPP is the higher stability of anthocyanin pigments during process resulting from lowering of pH after application of carbon dioxide to the product. Moreover, the pressures used in this process is minimum tenfold lower than at HPP what can positively influenced on the condensation processes, limited under pressures below 100 MPa [106].

### 5.3. High Pressure Homogenization (HPH)

It is well known that the presence of enzymes, oxygen, increasing pH and temperature, leads not only to the anthocyanins degradation, but can also cause their polymerization [92,115]. High pressure homogenization imposes mechanical stresses and then an inherent rise of temperature of the processed product [27,101]. Thus, there are indications that HPH and UHPH could cause anthocyanins content decrease. However Frank et al. [22] during HPH treatment at pressure 30–150 MPa of aqueous extract of bilberry, obtained almost constant anthocyanins concentration. Authors reported that in a solution of pH < 4, processed at the previously mentioned pressure range, no anthocyanins degradation due to fluid-mechanical stresses has been detected. Moreover, under these conditions anthocyanin molecules degradation was only temperature dependent. No thermal reduction was observed during high pressure treatment when rapid cooling after processing was applied. It was concluded that the HPH technique is a promising emerging method to provide best quality encapsulated and stabilized anthocyanin-rich emulsions.

Yu et al. [34] experiments with the UHPH treatment of mulberry juice also led to the finding that this kind of food preservation method can give good results in terms of anthocyanins retention. UHPH processing at 200 MPa with one and three passes and ascorbic acid addition to the matrix did not cause significant reduction in the content of cyanidin 3-glucoside (Cy-3-glc) and cyanidin 3-rutinoside (Cy-3-rut). Ascorbic acid in this case was necessary to prevent the oxidation of anthocyanins in juice. When no ascorbic acid was added, action of the peroxide or polyphenoloxidase and dissolved oxygen took place, resulting in significant Cy-3-glc and Cy-3-rut reduction, 38.8% and 33.2%, respectively. Not only ascorbic acid inhibits anthocyanins oxidation. Yu et al. [34] observed the protective role of phenolic acids in mulberry juice, because they are more prone to oxidation than anthocyanins. After one-pass UHPH processing of fresh mulberry juice with no ascorbic acid added, all five identified phenolic acids decreased from 10% to 35%. Further reduction to 30%–40% of initial content was observed when UHPH processing was continued to three passes, while anthocyanins content showed no further reductions.

The research of Karacam et al. [101] on the influence of cooling system during HPH on anthocyanins retention showed how important is this process. The authors subjected strawberry juice to HPH processing at 60 and 100 MPa with two or five passes. Cooling was not used after HP-valve nor after completion of the entire procedure. After 2 and 5 passes of juice through the homogenizer at 100 MPa, the final recorded temperature was 43.3 and 56.6 °C, respectively (data not shown for 60 MPa). Raised temperatures gave colour changes of the samples, i.a. drop of a* value (redness) what was connected with the reduction of anthocyanins level. At 100 MPa redness of juice decreased from 2 passes to 5 passes, interpreted as degradation of anthocyanins which are compounds mainly responsible for the red colour of strawberries. According to the authors the reduction of anthocyanins occurred as a result of elevated temperature and also may be related to the Maillard reaction, which products react with anthocyanins, thereby accelerating their degradation.

Ultra high pressure processing should be considered as a good pretreatment method of fruit puree containing anthocyanins before spray drying. According to the published results, homogenization of raspberry puree with 10% of water solution of arabic gum at pressure of 207 MPa with five passes and cooled coil gave excellent particle comminution and better coating of particles by arabic gum matrix, which manifested as better anthocyanins retention in encapsulated powders than in powders obtained without UHPH processing. The most important observation is that UHPH technique allowed to extract more anthocyanins from raspberry puree than a homogenizator working at ambient pressure [116].

Negative factor of HPH is the temperature increasing during homogenization which can influenced on the anthocyanins stability. The application of cooling system is generally enough for maintain temperature during HPH. There is no prove on the influence of the homogenization process on the degradation of anthocyanins. The biggest advantages of this process is the continuous operation mode and thus much higher efficiency compared to other techniques.

## 6. Traditional Thermal Processing (TP) vs. Pressure Techniques

Table 3 summarizes recently published results concerning the impact of temperature on fruit anthocyanins. Basically, TP preservation at temperature from 70 to 140 °C has a much greater effect on anthocyanins degradation compared to high pressure techniques. Even as short processing time as 2 min of thermal treatment of strawberry pulp at 70 °C caused significant decrease of total content of anthocyanins (20%) [65]. Increasing of temperature and time of treatment led to higher degradation of the pigments. To improve thermal stability of anthocyanins different approaches have been used, including the arabic gum addition to anthocyanins solutions. Arabic gum in an amount of 10 mg/mL increased the half-life of anthocyanins by 2.0 and 1.35 times at 80 and 126 °C, respectively [117].

## 7. Influence of High Pressure on the Anthocyanin Stability during Storage

### 7.1. High Pressure Processing (HPP)

Anthocyanins present in fruit and vegetable products subjected to HPP are not stable during storage. The changes of these pigments during storage occur through non-enzymatic as well as enzymatic reactions, due to partial tissue enzymes and microorganisms activity (Table 4). The 7 days storage studies at 20 and 30 °C of blackcurrants pressurized at 200–800 MPa for 15 min at room temperature showed that cyanidin-3-rutinoside and delphinidin-3-rutinoside were degraded, whereas cold storage at 4 °C did not influence anthocyanins [118].

The mechanism of enzymatic degradation of anthocyanins, presented in Figure 2, is connected with the incomplete inactivation of tissue enzymes like β-GLC, PPO and POD [74,119]. The HPP inactivation of PPO at 800 MPa at room temperature for 15 min was linked with the stability of pelargonidin-3-glucozide and pelargonidin-3-rutinoside during storage [74]. The significant role of β-GLC and POD in anthocyanin degradation during storage was confirmed by other authors [52,120]. Degradation of cyanidin-3-glucoside and cyanidin-3-sophoroside during prolonged storage (9 days) of pressurized raspberries were connected with a lower degree of inactivation of these enzymes [106].

Substrate specificity of β-glucosidase plays a significant role at the first step of anthocyanins degradation. Zabetakis et al. [119] noted higher loss of pelargonidin-3-glucoside compared with pelargonidin-3-rutinoside in HPP treated strawberries, at the same level of β-GLC residual activity, because of greater affinity of this enzyme towards glucose compared to rutinose. Enzyme degradation of anthocyanin pigments by β-GLC is mainly due to the loss of glucose leading to the formation of anthocyanidins (aglycon) affecting the red colour [3,52,84]. A similar trend was also described by Gimenez et al. [121] in strawberry jams.

Ascorbic acid is one of the important factors influencing the stability on anthocyanins pigments. This acid, apart from being an antioxidant, plays a significant role in the acceleration of anthocyanins degradation [118]. Ascorbic acid and anthocyanins diminish simultaneously in stored fruit products. The degradation of anthocyanins by ascorbic acid is induced indirectly by hydrogen peroxide formation during oxidation, therefore supplying a substrate for enzymatic reaction catalyzed by POD [1].

Anthocyanin losses can be reduced by storing of HPP treated samples at low temperatures.

Changes in the content of the anthocyanin pigments in strawberry puree during storage, described as the kinetic rate constant, were previous studied by Marszałek et al. [76]. Authors showed that kinetics rate constant generally increased with increasing the pressure of process from 300 to 600 MPa and ranged from 0.38 to 2.17 × 10^−2^ days^−1^ for sample stored at 6 °C. Authors connected this phenomenon with higher PPO residual activity at higher pressure which significalntly influence on the anthocyanins stability during storage. On the other hand Cao et al. [123] noted higher kinetic rate constant range from 0.056 to 0.505 months^−1^, calculated for anthocyanins in strawberry juice preserved at 600 MPa, 4 min and stored at cold and room temperature, respectively.

Different hypothesis have been postulated on the mechanism of degradation of anthocyanins during storage. The reduction of anthocyanins content in most cases was probably connected with oxidation processes, condensation with phenolic compounds, polymerization and enzyme degradation [105,125,127]. Garcia-Palazon et al. [74] observed that two different pelargonidins in raspberry were stable during storage, when PPO was inactivated. Zabetakis et al. [119] related anthocyanins degradation during storage with specificity of some substrates of β-glucosidase and anthocyanins. Cao et al. [123] found a loss of anthocyanins in cloudy strawberry juices, which was possibly due to the higher concentration of oxygen absorbed in pulp particles promoting the degradation. The stability of anthocyanins was also influenced by other fruit components, for instance by products of degradation of ascorbic acid and monosaccharides may accelerate anthocyanins degradation [109,127]. It is well known that during storage in some pressurized food enzymatic browning appears, but it is also observed that the addition of antioxidant such as ascorbic acid inhibits the browning effect and the activity of some enzymes such as POD is reduced [109]. Moreover, higher temperature of storage significantly accelerates all biochemical reaction. HPP facilitates the enzymatic and non-enzymatic reactions, because due to cell disruption substrates, ions and enzymes located in different compartments in the cell are liberated and can interact with each other.

Similarly to enzymatic browning, the non-enzymatic Maillard reaction is known to be affected by many factors, including temperature and pH [109].

### 7.2. High Pressure Carbon Dioxide (HPCD)

In the research on strawberry juice led by Marszałek et al. [85] samples were pressurized with carbon dioxide in a supercritical state for 30 min at 45 °C in 30 and 60 MPa. The experiments proved first order kinetics of anthocyanin degradation. Established half-life values were 22.4–28.6 days for 30 MPa treatment and 26.4–32.7 days for 60 MPa treatment. Changes in the content of the anthocyanin pigments in strawberry juice during storage after HPCD treatment, described as the kinetic rate constant, were reported by Marszałek et al. [85]. Authors showed that kinetics rate constant of decomposition of each monomer in strawberry juice stored at 6 °C increased from 0.021 to 0.031 days^−1^ with increasing pressure from 30 to 60 MPa. This phenomenon was connected with higher POD residual activity noted in juices treated by the lower pressure in HPCD treatment.

Storage research on blood orange juice treated with SCCD at 13 and 23 MPa and 36 °C has shown that anthocyanins were stable for 20 days. Subsequent degradation was connected with a rapid microbial growth in samples rather than with the activity of tissue enzymes [110].

Stability of anthocyanins in extracts of *Hibiscus sabdariffa* preserved by HPCD was comparable with non-treated samples; 14 weeks of storage caused less than a 10% decrease of the amount of pigments [111]. It should be noted that due to the method of food products preparation the activity of the endogenous enzyme is suspected to be negligible.

The research by Zou et al. [112] on mulberry beverage stored after treatment with HPCD at 55 °C, 15 MPa for 10 min showed significant losses of anthocyanins after 7 days at 25 °C and after 14 days at 4 °C, however no numerical values have been provided. The degradation of anthocyanins has been associated with the activity of polyphenol oxidases. The stability of anthocyanins found in muscadine grape juice treated by HPCD and stored for four weeks at 4 °C with no residual activity of this enzyme were 4.5 fold higher compared with juice with active enzyme [88]. Juices preserved by HPCD in the presence of oxidoreductases offered appreciable retention of the phytochemical variables that was attributed with decrease in dissolved oxygen and protection of non-anthocyanin phenolic compounds during refrigerated storage [88]. Some studies have shown that reversible changes in protein structure can occur following enzyme inactivation and may be influenced by processing conditions such as pressure, carbon dioxide, temperature, holding time and the subsequent conditions of storage [88]. Temperature of storage plays a crucial role in the rate of anthocyanins degradation during storage, since low temperature slow enzymatic reactions.

### 7.3. High Pressure Homogenization (HPH)

According to the authors best knowledge, there are no publications on the stability of anthocyanins as well as their kinetics of degradation in fruit and vegetable products during storage after HPH or UHPH processing.

## 8. Conclusions

The pressure-based food preservation methods are an interesting alternative for the traditional thermal processing techniques like pasteurization. They are a response for increasing customers demand on a microbiologically safe food with a high nutritional value and superior sensory quality. Anthocyanins are exceptionally important in this context, because they are both biologically active compounds and the main pigments for many fruits and vegetables, being at the same time quite heat-sensitive.

The HPP processing is already industrially used for preservation of various food groups, however it is the least potent among the three discussed pressure techniques in inactivation of tissue enzymes. Use of HPCD allows minimizing losses of anthocyanins during processing and storage, however application of carbon dioxide leads to the major technical problems; additionally carbon dioxide can significantly alter sample properties such as acidity. HPH is a relatively young method and its use is so far limited to the laboratory trials. Despite all their flaws high pressure techniques can be advantageous against heat treatment in retention of health promoting compounds such as anthocyanins.

Although the mechanisms involved in degradation of anthocyanins are complex, the whole process can accurately be described by a first order reaction kinetics. Literature data show that activation energy is not dependent on pressure of HPP process, however its values are significantly lower those calculated for TP.

The knowledge about impact of high pressures on the tissue enzymes is so far limited to the empirical observations of their activity. For the better description of phenomena occurring during pressurization further research, including the changes in tertiary and quaternary structures of proteins during processing, should be performed. According to authors best knowledge there are limited number of publications about influence of HPCD and HPH techniques on the anthocyanins and tissue enzymes kinetics degradation.

The research on the effects of high-pressure food processing methods should be continued as industry is constantly implementing these techniques, which can in many cases be a good substitute to traditional thermal preservation.

## Figures and Tables

**Table 1:** Effect of HPP on the stability of selected fruit and vegetable enzymes.

Enzyme	Source	Processing Conditions	Effect	Reference
PPO	Apples	300–700 MPa, 25 °C, 1 min	Activation at 300 MPa and inactivation at 700 MPa	[68]
	Avocados	Up to 900 MPa, 25–77 °C	Pressure increase caused a decrease in inactivation rate	[72]
	Onions	>700 MPa, 25 °C, 10 min	Activation, maximal at 500 MPa	[70]
	Pears	400, 25 °C, 10 min	Activation	[69]
	Pears	900 MPa, 25 °C	slight inactivation	[72]
	White grapes	700 MPa, 25 °C	inactivation	[72]
	Pineapples	200–600 MPa, 30–70 °C, 5–20 min	25% and 70% maximum inactivation at 600 MPa for 20 min and 30 and 70 °C, respectively	[67]
	Feijoas	200–600 MPa, 1–13 min, room temperature	Maximum 30% inactivation at 600 for 7 min	[66]
	Strawberries	200–600 MPa, 5–15 min, room temperature	Slight activation at 200 MPa, and up to 80% inactivation at 600 MPa for 15 min	[73]
	Red raspberries, strawberries	400–800 MPa, 18 and 22 °C	30% inactivation at 800 MPa after 15 min treatment in raspberries, total inactivation after 15 min at 600 and 800 MPa and after 10 min at 800 MPa in strawberries	[74]
	Strawberry puree	100–690 MPa, 24–90 °C, 5–15 min	24% inactivation at 690 MPa and 90 °C for 5 and 15 min	[63]
	Strawberries	300–600 MPa, 20–60 °C, 2–10 min	Maximum 29% inactivation at 600 MPa for 10 min and 60 °C	[62]
	Strawberries 3 cultivars	600 MPa, 20 °C, 5 min	20%–40% of inactivation depending on cultivar	[64]
	Strawberry puree with 10% of sugar	200–600 MPa, 40–80 °C, 2.5–10 min	Maximum 50% inactivation	[75]
	Strawberry pulp	400–600 MPa, 5–25 min, room temperature	51% inactivation at 600 MPa for 25 min	[65]
	Strawberry puree	50–400 MPa, 20–60 °C	60% inactivation under 250 MPa and 20 °C,	[61]
	Strawberry puree	300–500 MPa, 0–50 °C, 5–15 min	72% inactivation at 500 MPa, 15 min, 50 °C	[13]
	Strawberry puree	300–600 MPa, 50 °C, 15 min	58% and 41% of inactivation at 300 and 600 MPa, 15 min, 50 °C, respectively	[76]
	Spinach	700 MPa, 20 °C, 15 min	86% inactivation	[77]
	Mango pulp	400–600 MPa, 40–60 °C, 5–15 min	Up to 63% inactivation at the harshest conditions	[78]
	Blueberry	0.1–700 MPa, 30–80 °C	Up to 6 fold increase activity at 500 MPa and 30 °C, significant decrease at minimum 500 MPa and temperature over 76 °C	[79]
	Pumpkin puree	300–900 MPa, 60–80 °C, 1 min	Up to 60% inactivation at 900 MPa and 70 °C	[80]
	Cloudy apple juice	600 MPa, 3 min	Up to 50% inactivation	[81]
	Wild berries	200–600 MPa, room temperature, 5–30 min	Up to 3 fold increase activity at 200 MPa, 5 min and insignificant inactivation at harshest conditions	[82]
	Beet root	650 MPa, 3–30 min	10%–25% inactivation depending on time	[83]
	Peach	600 MPa, 20–100 °C, 3–5 min	68% inactivation at 20 °C for 5 min and 90% inactivation at 80–100 °C after 3 min	[84]
POD	Strawberries	400–800 MPa, 18 and 22 °C	11%–35% inactivation at 600 MPa for 15 min in strawberries	[74]
	Strawberry puree	100–690 MPa, 24–90 °C, 5–15 min	97% inactivation at 100 MPa and 90 °C for 15 min, 35% inactivation at the same pressure and the lowest temperature	[63]
	Strawberries	300–600 MPa, 20–60 °C, 2–10 min	Maximum 59% inactivation at 600 MPa for 2 min and 60 °C	[62]
	Strawberries of three cultivars	600 MPa, 20 °C, 5 min	40% inactivation	[64]
	Strawberry puree	50–400 MPa, 20–60 °C	50% inactivation under 230 MPa and 43 °C	[61]
	Strawberry pulp	400–600 MPa, 5–25 min, room temperature	74% inactivation at 600 MPa for 25 min	[65]
	Pineapples	200–600 MPa, 30–70 °C, 5–20 min	25% and 80% maximum inactivation at 600 MPa for 20 min and 30 and 70 °C, respectively	[67]
	Feijoas	200–600 MPa, 1–13 min, room temperature	Maximum 24% inactivation at 400 for 13 min and 600 MPa for 7 min	[66]
	Strawberry puree	300–500 MPa, 0–50 °C, 5–15 min	50% inactivation at 500 MPa, 15 min, 50 °C	[13]
	Strawberry puree	300–600 MPa, 50 °C, 15 min	31% and 83% inactivation at 300 and 600 MPa, 15 min, 50 °C, respectively	[76]
	Spinach	700 MPa, 20 °C, 15 min	77% inactivation	[77]
	Mango pulp	400–600 MPa, 40–60 °C, 5–15 min	Up to 67% inactivation at the harshest conditions	[78]
	Cloudy apple juice	600 MPa, 3 min	Up to 50% inactivation	[81]
	Wild berries	200–600 MPa, room temperature, 5–30 min	Up to 3 fold increase activity at 200 MPa, 5 min and insignificant inactivation at harshest conditions	[82]
	Beet root	650 MPa, 3–30 min	Up to 25% inactivation	[83]
	Peach	600 MPa, 20–100 °C, 3–5 min	26% inactivation at 20 °C for 5 min and 92% inactivation at 80–100 °C after 3 min	[84]
β-GLC	Red raspberries, strawberries	400–800 MPa, 18 and 22 °C	10% inactivation at 600 and 800 MPa for 15 min in raspberries, 50% and 60% reduction at 600 and 800 MPa for 15 min in strawberries	[74]
	Strawberry pulp	400–600 MPa, 5–25 min, room temperature	41% inactivation at 600 MPa for 25 min	[65]
	Strawberries	200–800 MPa, room temperature, 15 min	Increase of about 50% and 70% at 200 and 400 MPa, decrease of about 50% and 65% at 600 and 800 MPa.	[85]

**Table 2:** Effect of HPP on the anthocyanins stability.

Source	Anthocyanin Studied	Processing Conditions	Effect	Reference
Orange juice	Cy-3-glc	400–600 MPa for 15 min at 20 °C	99% retention of Cy-3-glc at 600 MPa	[1]
Pure anthocyanin	Cy-3-glc	200, 600 MPa at 25 °C and 70 °C for up to 6 h	Insignificant changes at 200 MPa and 70 °C, 25% loss of Cy-3-glc after 30 min at 600 MPa and 70 °C, 53% loss after 6 h at the same parameters	[106]
Blueberries	Total anthocyanins content	200–600 MPa, 5–15 min, 25 °C	Insignificant changes	[107]
Pomegranate juice	Total anthocyanins content	400–600 MPa, 25–50 °C, 5–10 min	Slight decrease progressing with increasing the pressure and temperature	[108]
Strawberry and wild berry mousses, pomegranate juice	Total anthocyanins content	500 MPa, 50 °C, 10 min for mousses, 400 MPa, 25 °C, 5 min for juice	90% retention in strawberry and wild berry mousses and 37% of losses in pomegranate juice	[104]
Strawberry pulps	Cy-3-glc Pg-3-glc Pg-3-rut	400–600 MPa, 5–25 min, room temperature	Insignificant changes of Cy-3-glc and Pg-3-glc, 6% loss of Pg-3-rut at 400 MPa, 10 min	[65]
Strawberries 3 cultivars	Cy-3-glc Pg-3-glc Pg-3-rut	600 MPa, 20 °C, 5 min	20%–28% losses depending on the strawberry cultivar	[64]
Strawberry and raspberry pastes and juices	Cy-3-glc Pg-3-glc Pg-3-ara Cy-3-soph Cy-3-rut	400–700 MPa, 20–110 °C, 20 min	Up to 23% changes at temperature below 80 °C and ca. 80% losses at temperature over 80 °C	[105]
Strawberry paste	Cy-3-rut	200–700 MPa, 80–110 °C, up to 50 min	Increasing the pressure accelerated degradation from 1.7 to 2.4 times (depending on the temperature), increasing the temperature accelerated degradation from 5.0 to 6.0 times (depending on the pressure)	[7]
Strawberries	Total anthocyanins content	300–600 MPa, 20–60 °C, 2–10 min	Insignificant changes	[62]
Strawberry puree	14 different anthocyanins compounds and their condensed pigments	100–400 MPa, 20–50 °C, 15 min	Insignificant changes	[109]
Strawberry puree	Cy-3-glc Pg-3-glc Pg-3-rut	300–500 MPa, 0–50 °C, 5–15 min	8% losses at 0 °C and 15% at 50 °C, insignificant influence of pressure and time	[13]
Strawberry puree	Cy-3-glc Pg-3-glc Pg-3-rut	300–600 MPa, 50 °C, 15 min	Up to 20% losses at 600 MPa	[76]

**Table 3:** Effect of thermal processing on the anthocyanins stability.

Source	Anthocyanin Studied	Processing Conditions	Effect	Reference
Pure anthocyanin	cyanidin-3-glucoside	70 °C for up to 6 h	5% loss after 30 min, 25% loss after 6 h	[106]
Strawberry pulp	cyanidin-3-glucoside, pelargonidin-3-glucoside, pelargonidin-3-rutinoside	70 °C, 2 min	20% loss of total anthocyanins	[65]
Strawberries	cyanidin-3-glucoside, pelargonidin-3-glucoside, pelargonidin-3-rutinoside	88 °C, 2 min	22%–25% loss of total anthocyanins	[64]
Strawberry and raspberry pastes and juices	cyanidin-3-glucoside, pelargonidin-3-glucoside, pelargonidin-3-arabinoside	80–140 °C, 20 min	Significant degradation of all monomer, at 140 °C almost total degradation of anthocyanins	[105]
Strawberry paste	cyanidin-3-glucoside	95–130 °C, up to 50 min	Increasing the temperature from 95 to 130 °C increased Cy-3-Glc degradation 15×	[7]
Strawberry puree	cyanidin-3-glucoside, pelargonidin-3-glucoside, pelargonidin-3-rutinoside	90 °C, 15 min	43% losses	[13]

**Table 4:** Effect of HPP on the anthocyanins stability during storage.

Source	Anthocyanin Studied	Processing/Storage Conditions	Effect	Reference
Raspberries	Cy-3-glc Cy-3-Soph	200–800 MPa, 18–22 °C, 15 min, Storage : 4, 20, 30 °C for 9 days	Greater stability at 800 MPa for Cy-3-glc and Cy-3-Soph at 4 °C of storage	[120]
Blackcurrants	Dp-3-rut Cy-3-rut	200–800 MPa, 18–22 °C, 15 min, Storage: 5, 20, 30 °C for 7 days	Greater stability at 600 MPa for Cy-3-rut and 800 MPa for Dp-3-rut at 5 °C of storage	[118]
Muscadine grape juice	Delphinidin-3, 5-diglucoside, Petunidin-3,5-diglucoside, Peonidin-3,5-diglucoside, Malvidin-3,5-diglucoside	400 and 550 MPa for 15 min, Storage: 25 °C for 21 days	28%–34% losses at 25 °C of storage	[122]
Orange juice	Cy-3-glc	400–600 MPa for 15 min at 20 °C, Storage : 4 and 20 °C for 10 days	93% and 89% retention at 600 MPa in juice stored at 4 and 20 °C	[1]
Strawberry and wild berry mousses, pomegranate juice	Total anthocyanins content	500 MPa, 50 °C, 10 min for mousses, 400 MPa, 25 °C, 5 min for juice, Storage: 4 and 25 °C for 72 days	35%–37% of losses for both products	[104]
Strawberries	Cy-3-glc Pg-3-glc Pg-3-rut	600 MPa, 20 °C, 5 min, Storage: 4 °C for 3 months	19%–25% retention after 3 month of storage	[64]
Strawberry cloudy and clear juices	Cy-3-glc Pg-3-glc Pg-3-rut	600 MPa, room temperature, 4 min, Storage: 4 and 25 °C for 6 months	30% and 7% losses in cloudy and clear juices	[123]
Strawberries	Pg-3-glc Pg-3-rut	200–800 MPa, 18–22 °C, 15 min, Storage: 4, 20, 30 °C for 9 days	Greatest stability after processing at 800 MPa; storage at 4 °C	[119]
Strawberry puree	Cy-3-glc Pg-3-glc Pg-3-rut	300–600 MPa, 50 °C, 15 min, Storage: 6 °C for 4 and 28 weeks for 300 and 600 MPa, respectively	fastest degradation after processing at 600 MPa compared to 300 MPa, half-life: 62 and 86 days, respectively for 600 and 300 MPa, the lowest stability of Pg-3-rut	[76]
Strawberry puree	Cy-3-glc Pg-3-glc Pg-3-rut	500 MPa, 50 °C, 15 min, Storage: 6 °C for 12 weeks	73% degradation of TCA, the lowest stability of Pg-3-rut	[124]
Strawberry	Total anthocyanins content	400 MPa, room temperature, 5 min, Storage: 4 and 25 °C for 45 days	33% and 57% degradation in samples stored at 4 and 25 °C	[125]
Bayberry juice	Cy-3-glc	400–600 MPa, room temperature, 10 min, Storage: 4 and 25 °C for 25 days	8% degradation in samples stored at 4 and significantly higher in samples stored at 25 °C	[126]
Pomegranate juice	Total anthocyanins content	300–400 MPa, room temperature, 2.5–25 min, Storage: 4 °C for 90 days	25% degradation	[127]
Aronia juice	-	200–600 MPa, room temperature, 15 min, Storage 4 °C for 80 days	Ca. 40% degradation at 600 MPa	[128]
